# Awareness of hepatitis C prevention and treatment and high-risk behaviors among the general population in Anhui Province: a cross-sectional study

**DOI:** 10.3389/fpubh.2025.1534169

**Published:** 2025-03-12

**Authors:** Seying Dai, Ziwei Wang, Qian Guo, Gan Tang, Qisheng Guo, Jin Zhang, Yinguang Fan

**Affiliations:** ^1^Anhui Provincial Center for Disease Control and Prevention, Hefei, Anhui, China; ^2^Department of Epidemiology and Health Statistics, School of Public Health, Anhui Medical University, Hefei, Anhui, China

**Keywords:** hepatitis C, awareness, high-risk behaviors, public health, Anhui Province

## Abstract

**Background:**

The World Health Organization (WHO) set the goal of “eliminating viral hepatitis as a major public health threat by 2030” in 2016. In 2021, the National Health Commission of China (NHCC), issued an action plan to help achieve the WHO’s goal of eliminating the Hepatitis C virus by 2030. Therefore, the primary objective of this study was to investigate the awareness of knowledge of hepatitis C prevention and treatment and high-risk behaviors among the general population of Anhui Province.

**Methods:**

Stratified sampling method had been used to select participants to conduct a survey from June 2021 to September 2021 in Anhui Province. Multivariate logistic regression model was used to reveal the influencing factors of participants’ awareness and the self-selected high-risk behaviors of HCV infection.

**Results:**

The crude and standard awareness rates of hepatitis C were 56.12% (95% *CI*: 54.15–58.11%) and 53.74% (95% *CI*: 53.72–53.75%), respectively. Among the 2,423 participants, 83.2% knew that blood or blood products can lead to hepatitis C infection, but only 44.2% knew that people infected with HCV can look healthy. Multivariate logistic regression model analysis showed that age group, education level, and geographic location were the important factors influencing hepatitis C awareness. In the last year, 1,113 people (45.9%) reported that they had high-risk behaviors for hepatitis C infection. Multivariate logistic regression model analysis revealed that hepatitis C awareness, gender, marital status, and geographic location were the important factors influencing the self-selected high-risk behaviors.

**Conclusion:**

The findings indicated that the general population in Anhui Province has low awareness of HCV prevention and treatment and a certain degree of history of high-risk behavior for hepatitis C. In the future, more information and health education on hepatitis C is needed, with particular attention to the older adult, those with low education levels, and the central and southern regions of Anhui Province. We also should strengthen the education of females and married, divorced or widowed individuals to recognize and avoid high-risk behaviors for hepatitis C in their lives. By narrowing the gap between knowledge and behavior, we can contribute to the goal of eliminating hepatitis C by 2030.

## Background

Hepatitis C virus (HCV) infection, which most patients are asymptomatic and must be tested to detect, is an urgent public health issue worldwide. Following acute infection, spontaneous eradication of the virus may occur in as many as 45% of young healthy patients who develop a strong immune response. However, most infected individuals are unable to clear the virus, which can lead to a chronic infection that may progress to cirrhosis or hepatocellular carcinoma increasing the disease burden (1). The World Health Organization (WHO) estimates that globally around 58 million people are living with chronic hepatitis C, about 1.5 million new cases of HCV infections each year, as well as approximately 290,000 deaths in 2019 due to hepatitis C (2). In China, about 7.6 million people are infected with chronic HCV, one of the highest numbers in the world (3). The Global Health Sector Viral Hepatitis Strategy 2016–2021, adopted at the 69th World Health Assembly in 2016, set the overall goal of “eliminating viral hepatitis as a major public health threat by 2030” (2).

With the advent of generally well-tolerated direct-acting antivirals (DAAs), the perception of HCV has transformed, HCV is now widely viewed as a curable disease, with a 95% cure rate (4). More importantly, DAAs can dramatically stop the progression of the disease toward liver cirrhosis and reduce the risk of hepatocellular carcinoma development (5–7). China implemented a series of national policies to improve the accessibility and affordability of DAAs, including the development of DAAs and the inclusion of DAAs in health insurance (8). However, a vast number of hepatitis-infected individuals are still devoid of available and accessible treatment services (9). The WHO estimated that the cumulative treatment rate of HCV infection in China was only 1% by 2020 (10), and the diagnosis rate for people with hepatitis C was only 25% (11).

The level of individuals’ knowledge about the disease significantly influences their behaviors and attitudes, as higher levels of knowledge were related to a larger likelihood of exhibiting positive attitudes (12). The awareness of HCV has previously been evaluated in various countries. Studies have been done in specific populations such as injecting drug users (13), university students (14), men who have sex with men (MSM) (15), and health care workers (16), but research on the knowledge level of the general population is limited. Moreover, the data of surveys on the awareness of hepatitis C knowledge was inadequate in China, and it is mostly concentrated in local cities (17, 18), lacking provincial-level data to assess the general population’s awareness of hepatitis C. In light of this, we conducted a hepatitis C awareness survey in Anhui Province to fill the gap in this area.

Hepatitis C, similar to hepatitis B, is primarily transmitted through percutaneous blood contact, while less commonly, it can also be transmitted through mother-to-child transmission and sexual intercourse (1). Certain high-risk behaviors may cause people to be exposed to diseases and spread them to others. It had been shown that high-risk behaviors are associated with HCV seroprevalence (19). In a study on HCV, it was found that the awareness of transmission routes was relatively high, while the awareness of specific behaviors associated with transmission, such as eyebrow trimming, tattooing, ear piercing, and other traumatic cosmetic procedures, was relatively low (20). These behaviors are considered risky for HCV transmission today.

The Healthy China Action (2019–2030) released in 2019 and the Public Health Treatment Action Plan for Hepatitis C Elimination (2021–2030) released in 2021 by the National Health Commission of China (NHCC) and other multi-departmental organizations, aim to contribute to the WHO’s goal of eliminating the hepatitis C virus by 2030 (21, 22). It is urgently needed to conduct a survey to assess the awareness of HCV among general people. Hence, the objective of this study was to investigate the awareness of knowledge of hepatitis C prevention and treatment, the occurrence of high-risk behaviors and their influencing factors in the general population of Anhui Province.

## Methods

### Study design and participants

Based on previous studies (17, 18), the HCV awareness rate of the general population in city China ranged from 20.36 to 60.2%, and the mean value was taken as 40.28%, which led to the estimation of the minimum sample size needed for this survey. The formula for calculating sample size is as follows: $n=de\mathrm{ff}*z_{\alpha /2}^{2}*\frac{P*\left(1-P\right)}{\delta^{2}}$ , (where the awareness rate was *p* = 0.40, the allowable error was *δ* = 0.063, the stratified sampling design efficiency was *deff* = 1.50, significance level was *α* = 0.05) the sample size required for each city is calculated as *n* = 337. A total of 2,022 people needed to be surveyed in 6 cities, taking into account the 10% of the non-response rate, and 2,247 participants eventually were required.

The study was conducted from June 2021 to September 2021. Subject inclusion criteria were (1) 15 years of age or older; (2) rural and urban residents; (3) 6 months and more of residence in the survey area; and (4) voluntary consent to participate. The method of stratified random sampling was as follows: firstly, Anhui Province was divided into southern, central and northern regions according to geographical location, and then two cities were randomly selected using simple random sampling method with the help of random number from each of the three regions (southern: Xuancheng city and Anqing city; central: Hefei city and Ma’anshan city; northern: Chuzhou city and Bozhou city), and then, one county or district was selected randomly by simple random sampling method with the help of random number from each of these six cities, and finally, Jing County, Mengcheng County, Chaohu County, Tianchang County, Daguan District, and Yushan District were selected as the investigation site. Four subdistricts (townships) were randomly selected from each county or district with simple random sampling method, then, one neighborhood committee (village committee) was chosen randomly from each subdistrict (township), for a total of 24 neighborhood committees (village committees). Finally, 94–104 general people were randomly selected from each neighborhood committee (village committee) for the survey, and the total number of people expected to be surveyed ranged from 2,256 to 2,496.

### Questionnaire

The questionnaire obtained from the Chinese Center for Disease Control and Prevention (CDC) was conducted by trained investigators following uniform standards and requirements for face-to-face, one-to-one questionnaire surveys. The questionnaire was composed of three sections. The first section focused on sociodemographic characteristics which included six items: survey area, gender, age, marital status, residence address, ethnic, and education level. The second section was the knowledge of HCV prevention and treatment which included eight items. One item asked whether a person who looks healthy carries HCV, while the remaining seven items covered various aspects of HCV, such as the transmission of HCV infection, whether cause cirrhosis or liver cancer, and whether Hepatitis C can be curable. Each item provided three response options (“True,” “False,” and “Don’t know”). Participants received points for correct responses, and their total knowledge score was calculated by summing these points, resulting in a range of 0–8. Participants whose total score was equal to or more than 6 were considered aware of hepatitis C prevention and treatment. The correct answer of section two will be told to the participants as soon as the whole questionnaire was finished. The third section was the high-risk behaviors among participants of HCV infection which included seven items. One item was focused on the risk of contracting hepatitis C, while the other six items explored the occurrence of high-risk behaviors in the past year. The Cronbach’s *α* coefficient of the questionnaire was 0.853, indicating a high degree of internal consistency, and the split-half reliability was 0.799.

### Definition of key variables

1. High-risk behaviors of hepatitis C infection: those who had any one or more of the above six high-risk behaviors of hepatitis C infection were defined as having a history of high-risk behaviors of hepatitis C. In this study, high-risk behaviors of hepatitis C infection can be divided into two categories: self-selected high-risk behaviors are defined as non-avoidable actions subjectively chosen based on personal life needs, including traumatic beauty or medical treatment such as liposuction, eyebrow tattooing, ear piercing, pedicure in informal medical institutions such as the street or small shops, tooth extraction/filling/cleaning in individual clinics, sharing syringes with others, commercial sexual behavior. Not self-selected high-risk behavior are defined as unavoidable diagnostic or therapeutic actions required due to medical reasons during treatment, including endoscopy examination (such as gastroscopy, colonoscopy, laparoscopy, hysteroscopy, fiberoptic bronchoscopy, etc.), blood transfusion or blood products received;
2. Awareness of knowledge of hepatitis C prevention and treatment: participants whose total score was equal to or more than 6 were defined as aware, otherwise was unaware;
3. The correct rate of each question of hepatitis C related knowledge: the correct answer to the relevant knowledge question divided by the total number of people who answered the question ×100%;
4. The standardized rate of hepatitis C related knowledge: weighted calculation based on the age composition of the population of Anhui Province in 2021 (23).

### Statistical analysis

The data were entered into a database using EpiData V.3.2 by two researchers independently to ensure accuracy. Statistical analysis was performed through SPSS V.23.0. Qualitative data was described using frequency. To compare differences between groups, the Chi-squared test was used. Multivariate logistic regression model was used to reveal the influencing factors of participants’ awareness of knowledge of hepatitis C prevention and treatment and influencing factors of the high-risk behaviors of HCV infection. *p <* 0.05 were considered statistically significant.

## Results

### Basic characteristics of study participants

A total of 2,462 questionnaires were distributed and 2,423 valid questionnaires were collected, resulting in a valid response rate of 98.4%. Participant characteristics are summarized in Table 1. The majority of participants were Anhui residents (98.7%) with an average age of 40.5 ± 13.7 years; 1,228 (50.7%) were male and 1,195 (49.3%) were female. Among the participants, 1,896 (78.3%) were married, 474 (19.6%) were single, and 53 (2.2%) were divorced or widowed. The major portion of participants were Han (99.3%), survey area in urban (51.9%), the majority of them had a middle school education level (32.4%), followed by high school or technical secondary school (28.8%), college degree or above (20.2%), and primary school and below (18.6%). Geographically, 33.6% were from northern Anhui, 33.4% from central Anhui, and 33.0% from southern Anhui (Table 1).

**Table 1:** Baseline characteristics of study participants (*n* = 2,423).

Characteristics	*n* (%)
Survey area	
Urban area	1,257 (51.9)
Rural area	1,166 (48.1)
Gender	
Male	1,228 (50.7)
Female	1,195 (49.3)
Age group (years)	
15–34	913 (37.7)
35–54	1,073 (43.3)
≥55	437 (18.0)
Marital status	
Single	474 (19.6)
Married	1,896 (78.3)
Divorced or widowed	53 (2.2)
Residence address	
Anhui Province	2,391 (98.7)
Other Provinces	32 (1.3)
Ethnic group	
Han	2,405 (99.3)
Other groups^a^	18 (0.7)
Education level	
Primary school and below	451 (18.6)
Middle school	785 (32.4)
High school or technical secondary school	698 (28.8)
College degree or above	489 (20.2)
Geographical location	
Northern	815 (33.6)
Central	808 (33.4)
Southern	800 (33.0)

a Other groups are those recognized by China as ethnic minorities other than the Han ethnic group.

### Knowledge status of HCV among the general population

The awareness rate of hepatitis C was standardized according to the age composition of the Anhui population in 2021. The crude and standard awareness rates of hepatitis C were 56.12% (95% *CI*: 54.15–58.11%) and 53.74% (95% *CI*: 53.72–53.75%), respectively (Table 2). Among the 2,423 participants, 83.2% knew that blood or blood products can lead to hepatitis C infection, and 80.4% knew that HCV infection can occur when sharing needles with HCV carriers. 66.6% knew that hepatitis C can cause cirrhosis and/or hepatocellular carcinoma. In contrast, only 44.2% knew that people infected with HCV can look healthy and about half of the participants were unknown that HCV infection can be cured (Table 2).

**Table 2:** Knowledge statements about HCV prevention and treatment^b^ (*n* = 2,423).

Item no.	Item	Responses		
		Yes *n* (%)	No *n* (%)	Do not know *n* (%)
1	Can a person who looks healthy carry HCV?	1,072 (44.2)^c^	605 (25.0)	746 (30.8)
2	Can HCV be transmitted by shaking hands or eating with HCV-positive individuals?	595 (24.6)	1,325 (54.7)^c^	503 (20.7)
3	Can a person be infected with HCV by sex with HCV-positive individuals?	1,645 (67.9)^c^	237 (9.8)	541 (22.3)
4	Can a person be infected by HCV when sharing needles with HCV carriers?	1,947 (80.4)^c^	131 (5.4)	345 (14.2)
5	Can a person be infected with HCV by transmitting blood or blood products?	2,015 (83.2)^c^	70 (2.9)	338 (13.9)
6	Can a person be infected with HCV by tattoos/tattooed eyebrows and ear piercing?	1,565 (64.6)^c^	250 (10.3)	608 (25.1)
7	Can Hepatitis C cause cirrhosis and/or hepatocellular carcinoma?	1,615 (66.6)^c^	145 (6.0)	663 (27.4)
8	Can Hepatitis C be curable?	1,331 (54.9)^c^	272 (11.2)	820 (33.9)

b Values are given as number (percentage) unless indicated otherwise.

c Indicates correct responses.

### Factors associated with awareness of hepatitis C knowledge

Significant differences in awareness rates were found among different ages, marital status, education levels, and geographical location (Table 3). More than half of the participants aged 55 and above were unaware about hepatitis C prevention and treatment (*p <* 0.001). In terms of marital status, knowledge of hepatitis C prevention and treatment was more common among single people (*p* = 0.005). With respect to different geographic locations in Anhui Province, the highest awareness rate was found in the northern region of Anhui Province (*p <* 0.001). However, no statistically significant differences were found in hepatitis C knowledge by gender and survey area, but there was a trend toward increased knowledge of hepatitis C prevention and treatment with increasing education level and it was statistically significant (*p <* 0.001) (Table 3).

**Table 3:** Association of awareness with characteristics of study participants (*n* = 2,423).

Characteristics	Total (*n*)	Awareness *n* (%)	*χ* ^2^	*p*-value
Survey area			1.217	0.270
Urban area	1,257	719 (57.2)		
Rural area	1,166	641 (55.0)		
Gender			0.011	0.918
Male	1,228	688 (56.0)		
Female	1,195	672 (56.2)		
Age group (years)			49.263	<0.001
15–34	913	580 (63.5)		
35–54	1,073	590 (55.0)		
≥55	437	190 (43.5)		
Marital status			10.489	0.005
Single	474	297 (62.7)		
Married	1,896	1,036 (54.9)		
Divorced or widowed	53	27 (50.9)		
Residence address			3.265	0.071
Anhui Province	2,391	1,337 (55.9)		
Other Provinces	32	23 (71.9)		
Ethnic Group			0.183	0.669
Han	2,405	1,349 (56.1)		
Other groups	18	11 (61.1)		
Education level			69.602	<0.001
Primary school and below	451	198 (43.9)		
Middle school	785	401 (51.1)		
High school or technical secondary school	698	432 (61.9)		
College degree or above	489	329 (67.3)		
Geographical location			83.023	<0.001
Northern	815	529 (64.9)		
Central	808	484 (59.9)		
Southern	800	347 (43.4)		

The multivariate logistic regression analysis showed results in Table 4, compared to participants aged 15–34, participants aged 35–54 no difference (*OR* = 0.911, 95% *CI*: 0.745–1.113, *p* = 0.362), and participants above 55 (*OR* = 0.632, 95% *CI*: 0.485–0.824, *p* = 0.023) were less aware. Among those with middle school (*OR* = 1.455, 95% *CI*: 1.132–1.871, *p* = 0.003), high school or technical secondary school (*OR* = 2.341, 95% *CI*: 1.770–3.097, *p* < 0.001) and college degree or above (*OR* = 3.139, 95% *CI*: 2.274–4.333, *p* < 0.001) were more likely to have a high awareness rate, compared with Primary school and below. In addition, compared to the northern Anhui Province, the awareness rate was lower in the central (*OR* = 0.633, 95% *CI*: 0.510–0.785, *p* < 0.001) and southern (*OR* = 0.322, 95% *CI*: 0.259–0.400, *p* < 0.001) Anhui Province (Table 4).

**Table 4:** Multivariate logistic regression model of awareness of hepatitis C knowledge (*n* = 2,423).

Characteristics	*β*	$s_{\bar{x}}$	*OR* (95%*CI*)	*p*-value
Age group (years)				
15–34			1.000	
35–54	−0.094	0.103	0.911 (0.745–1.113)	0.362
≥55	−0.319	0.140	0.632 (0.485–0.824)	0.023
Education level				
Primary school and below			1.000	
Middle school	0.375	0.128	1.455 (1.132–1.871)	0.003
High school or technical secondary school	0.851	0.143	2.341 (1.770–3.097)	<0.001
College degree or above	1.144	0.164	3.139 (2.274–4.333)	<0.001
Geographical location				
Northern			1.000	
Central	−0.458	0.110	0.633 (0.510–0.785)	<0.001
Southern	−1.133	0.110	0.322 (0.259–0.400)	<0.001

### High-risk behaviors of HCV among the general population

**Figure 1:**
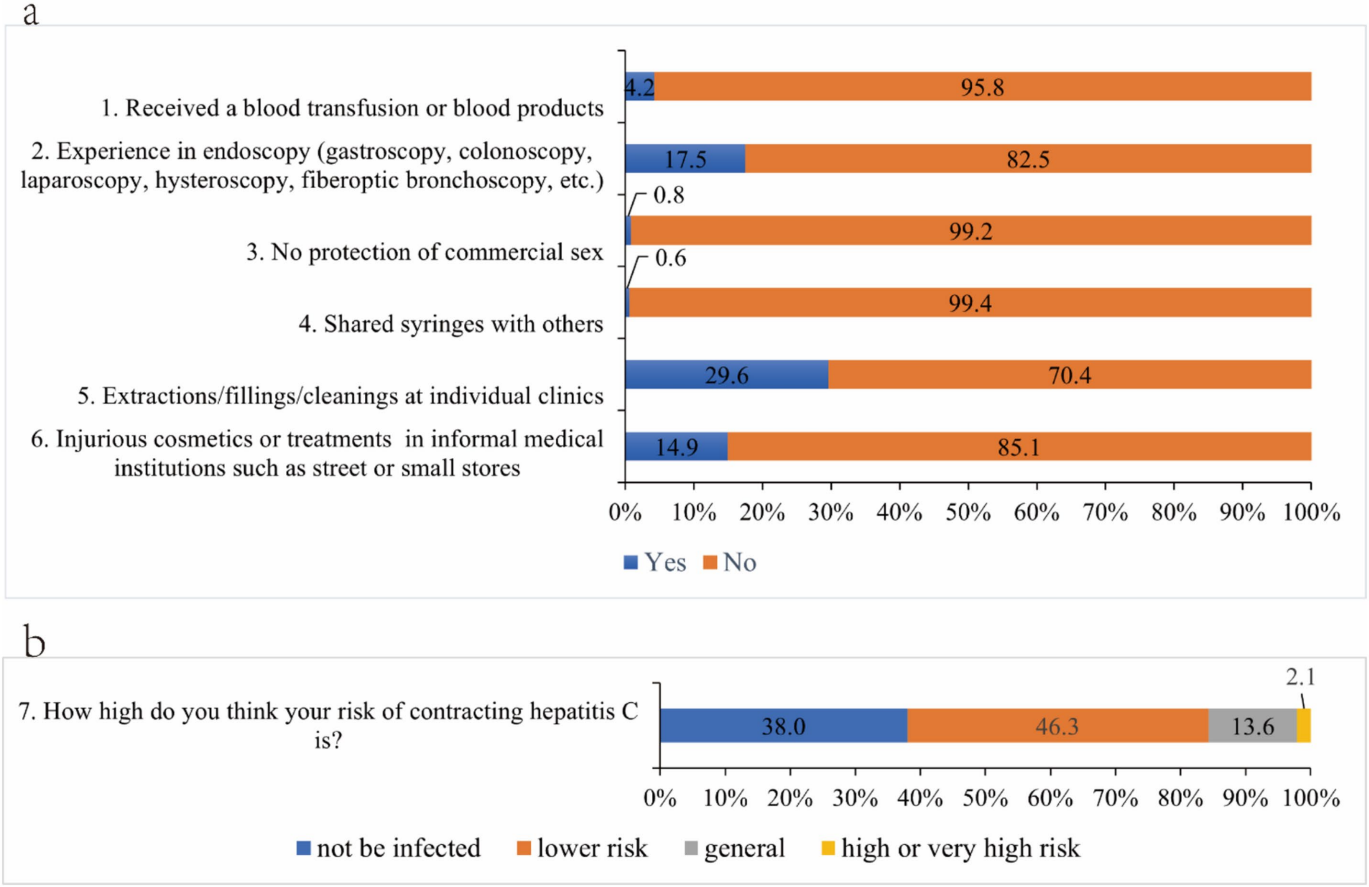
The occurrence of hepatitis C high-risk behaviors **(a)** and risk perception **(b)** among participants.

Among the 2,423 participants, 1,113 (45.9%) reported that they ever had high-risk behaviors of hepatitis C infection in the past year, among which the number of participants who had shared syringes with others was the least (0.6%), and the number of participants who had the experience of tooth extraction, filling and cleaning in private clinics was the biggest (29.6%) (Figure 1a) 921 (38.1%) participants believed that they would not be infected with HCV, and 50 (2.1%) believed they were at high or very high risk (Figure 1b). Among the high-risk behaviors of hepatitis C infection, 703 (29.0%) participants had one high risk behavior, 313 (12.9%) participants had two high risk behaviors, 81 (3.3%) participants had three high risk behaviors, and 16 (0.7%) participants had four or more high risk behaviors (Table 5). Among the four self-selected high-risk behaviors, 892 (36.8%) participants had at least one of them.

**Table 5:** Frequency of high-risk behaviors for hepatitis C (*n* = 2,423).

Frequency of high-risk behaviors for hepatitis C infection^d^	*n*	%
0	1,310	54.1
1	703	29.0
2	313	12.9
3	81	3.3
4	14	0.6
5	2	0.1

d None of the participants had six high-risk behaviors for hepatitis C infection.

### Factors associated with self-selected high-risk behaviors for hepatitis C

**Figure 2:**
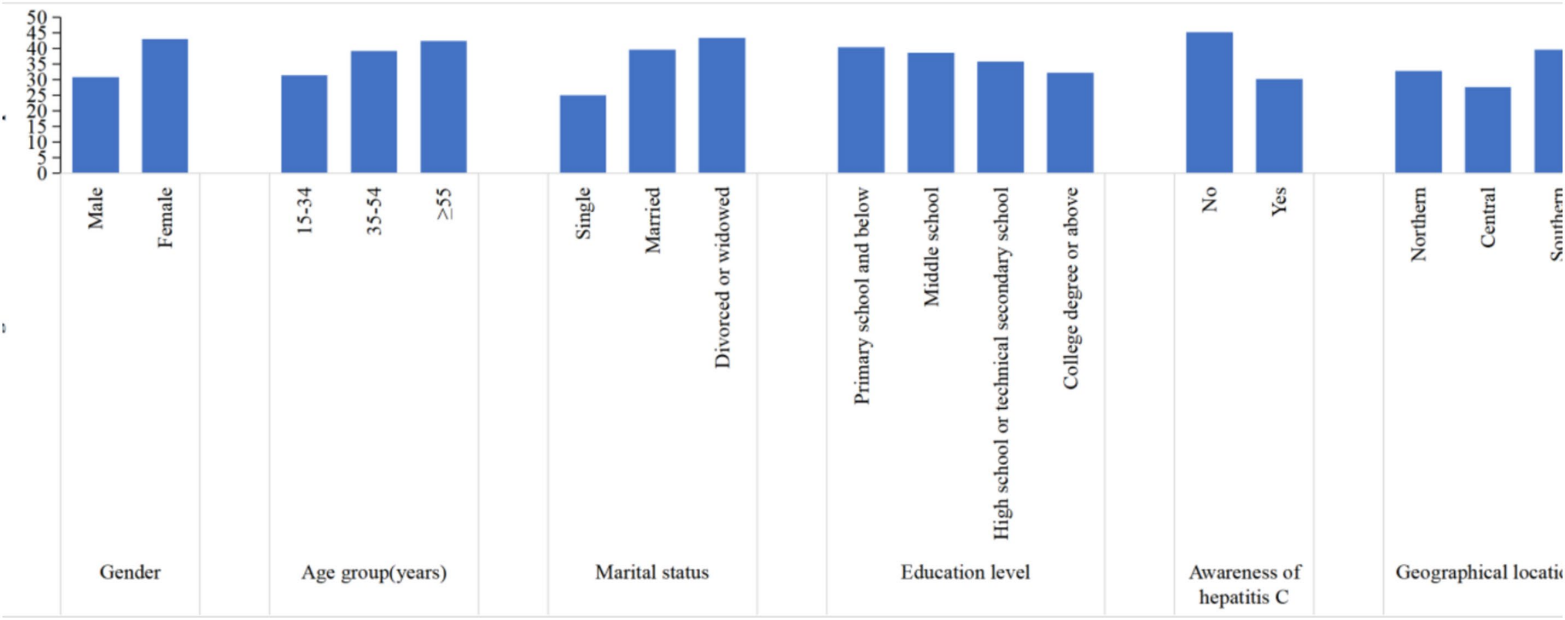
Occurrence of self-selected high-risk behaviors for hepatitis C significantly associated in univariate analyses.

Significant differences in the occurrence of self-selected high-risk behaviors were found by gender, age, marital status, educational level, whether they were aware of hepatitis C prevention and treatment and geographical location (Supplementary Table 1 and Figure 2). In this analysis of the self-selected high-risk behaviors survey, self-selected high-risk behaviors were found to be more prevalent in the female participants (*p* < 0.001). With increasing age, the occurrence of self-selected high-risk behaviors tended to increase and was statistically significant (*p* < 0.001). In terms of marital status, the self-selected high-risk behaviors were more common among the married and divorced or widowed populations (*p* < 0.001). Moreover, significant differences were observed in the occurrence of self-selected high-risk behaviors based on education level, with the highest occurrence of self-selected high-risk behaviors in primary school and below (*p* = 0.043). Furthermore, participants who were unaware of HCV infection prevention and treatment demonstrated a significantly higher occurrence of self-selected high-risk behaviors (*p <* 0.001). Significant differences in the occurrence of self-selected high-risk behaviors were also found in different geographic locations in Anhui Province, with the highest occurrence in the southern regions (*p <* 0.001).

The multivariate logistic regression model analysis showed results in Table 6, compared to male participants, participants who were female (*OR* = 1.693, 95% CI: 1.426–2.008, *p* < 0.001) were more likely to engage in self-selected high-risk behaviors. Additionally, married (*OR* = 1.833, 95% *CI*: 1.455–2.310, *p* < 0.001) and divorced/widowed (*OR* = 2.221, 95% *CI*: 1.224–4.030, *p* = 0.009) were significantly associated with the self-selected high-risk behaviors, compared with single. Being aware of hepatitis C knowledge (*OR* = 0.563, 95% *CI*: 0.473–0.670, *p* < 0.001) was a significant influence of reducing the occurrence of self-selected high-risk behaviors. Compared to the northern region of Anhui Province, the incidence of self-selected high-risk behaviors is lower in the central region (*OR* = 0.784, 95% *CI*: 0.634–0.970, *p* = 0.025), while the rate is higher in the southern region (*OR* = 1.355, 95% *CI*: 1.099–1.671, *p* = 0.005) (Table 6).

**Table 6:** Multivariate logistic regression model of self-selected high-risk behaviors (*n* = 2,423).

Characteristics	*β*	$s_{\bar{x}}$	*OR* (95% *CI*)	*p*-value
Gender				
Male			1.000	
Female	0.526	0.087	1.693 (1.426–2.008)	<0.001
Marital status				
Single			1.000	
Married	0.647	0.119	1.833 (1.455–2.310)	<0.001
Divorced/widowed	0.798	0.304	2.221 (1.224–4.030)	0.009
Awareness of hepatitis C				
No			1.000	
Yes	−0.575	0.089	0.563 (0.473–0.670)	<0.001
Geographical location				
Northern			1.000	
Central	−0.243	0.108	0.784 (0.634–0.970)	0.025
Southern	0.304	0.107	1.355 (1.099–1.671)	0.005

## Discussion

The primary objective of this study was to investigate the awareness of knowledge of hepatitis C prevention and treatment and the occurrence of high-risk behaviors among the general population in Anhui Province, China. Results indicated that the awareness of hepatitis C knowledge in the general population of Anhui Province was low compared with previous studies (24, 25), and had a certain degree of history of high-risk behavior for hepatitis C. Significant heterogeneity in self-selected high-risk behaviors was observed among participants with different characteristics. There is need to strengthen prevention and awareness of Hepatitis C, especially among female groups and married, divorced or widowed groups.

The total awareness rate of HCV prevention and treatment among our study participants in this survey was low, with only 56.1% of the participants being aware. Previous studies conducted in Ethiopia, and Guangxi found that 60.9% (24) and 69.25% (25) had good knowledge of hepatitis C. The country introduced DAAs with high cure rates in 2017 and issued a goal of eliminating public health harm from hepatitis C in 2019. However, there are still significant gaps in awareness and treatment of hepatitis C a vast number of the general population. In this study only 54.9% of participants believed that hepatitis C can be cured. A study of a Saudi Arabian population reported that only 47.3% of the study participants knew that the HCV was curable (26). Lack of awareness of a cure for hepatitis C may lead to fear of the disease among the general population, which in turn increases social prejudice and discrimination against patients. At the same time, infected patients may thus delay seeking medical treatment, increasing the risk of liver damage. Therefore, increasing public awareness of the curability of hepatitis C is essential for early intervention, reducing disease transmission and alleviating the healthcare burden on society. Although the result found that most participants knew that they can get hepatitis C by blood transfusion with HCV, only half (54.7%) knew that they cannot infect HCV by daily contact with a hepatitis C patient. These misconceptions may lead to community marginalization and discriminated against of HCV patients (27, 28). Therefore, a wide spread of accurate knowledge is important not only to prevent HCV transmission, but also to the quality in life of HCV patients. Remarkably, only 44.2% knew that people infected with HCV can look healthy, which may impact the proactive screening of high-risk individuals for hepatitis C, which in turn could lead to further spread of the disease. This reminds that we still need to strengthen our hepatitis C health education campaigns to reduce the risk of HCV transmission and promoting Hepatitis C awareness.

A study had shown that the awareness rate decreases with age increased (29). In the present study, the group aged 55 years and above had a lower awareness rate, which may be related to factors such as education levels, opportunities for science education and awareness in different age groups. Furthermore, in line with other studies, this study also showed that education level affects HCV awareness levels (7, 9, 30, 31). Highly educated people have been educated about hepatitis C protection at every stage of their studies and can obtain knowledge about HCV protection through newspapers, books and media. Compared with those with low education levels, they had a wide range of access and high acceptance of hepatitis C prevention and treatment knowledge. Additionally, due to social issues such as the imbalance between urban and rural development (32), residents in rural areas have access to fewer educational resources compared to those in urban areas. Therefore, future health education should be focused on the receptiveness of people with low education levels and residents in rural areas. The research results showed that the awareness rate in the central and southern regions of Anhui is lower compared to the northern region. This may be related to the higher HIV prevalence in the northern part of Anhui Province (33), where health education on related infectious diseases has been more extensively conducted. Therefore, in future hepatitis C prevention and treatment campaigns, Anhui should strengthen public education efforts in the central and southern regions to improve the overall level of hepatitis C knowledge across the province.

In this survey, only 2.1% of the participants considered themselves at high or very high risk of infection, but we found that nearly half of the participants had a history of high-risk behaviors in the six high-risk behaviors and 36.8% in the four self-selected high-risk behaviors, which indicated that a certain degree of high-risk behaviors occurred in Anhui Province, and this result was comparable to the results of previous study on the 48% occurrence of high-risk behaviors in Guangxi (25). The results showed that most people in Anhui Province are not fully aware of the potential risks of their behaviors, which also suggested that health education about high-risk behaviors and their consequences may not be widespread. This underscores the need to strengthen health education and raise public awareness of high-risk behaviors and their potential consequences. Moreover, we found a correlation between gender and the occurrence of self-selected high-risk behaviors for hepatitis C. This finding was closely related to another article published in 2020 on the hepatitis B virus among college students at a university in Tianjin, which noted statistically significant differences in the occurrence of damaging cosmetic behaviors such as eyebrow tattooing and ear piercing among students of different gender (34). The results suggested that the probability of high-risk behavior was higher among females, and therefore the probability of hepatitis C infection in females may increase in the future. However, some previous studies have found that males have a significantly higher rate of HCV antibody positivity than females (35, 36). When it comes to future health promotion, targeting females cannot be neglected. In addition, public education for privately operated beauty, hairdressing and dental clinics and other public places. Staff in places with blood exposure should establish the concept of prevention, eliminate the reuse of disposable medical supplies; standardize aseptic operations, sterilize medical devices thoroughly, and ensure the cleanliness and safety of instruments, the environment, and hands.

Self-selected high-risk behaviors were more likely to occur in married and divorced/widowed than single. Previous study had demonstrated that unmarried individuals were less likely to be infected with the HCV (37). Those with unmarried marital status also may be more concerned about their and the next generation’s reproductive health due to their childbearing age. Therefore, the occurrence of self-selected high-risk behaviors was lower among single people. Furthermore, our findings indicated that an important factor influencing the occurrence of self-selected high-risk behaviors for HCV was additionally knowledge of HCV prevention and treatment. The probability of engaging in self-selected high-risk behaviors was slightly higher for those who were unaware of hepatitis C prevention and treatment. However, previous studies have showed no difference in the occurrence of self-selected high-risk behaviors for hepatitis C between those who were aware of the knowledge of prevention and treatment and those who were not (25, 38), and separated knowledge from behavior existed, but in this study showed a statistically significant association. The heterogeneity of these results suggested that the importance of avoiding high-risk behaviors should be more emphasized in future publicity and education. Moreover, it is more important to carry out a timely assessment of residents’ awareness, attituded and behavior toward hepatitis C to effectively play the role of publicity and education and maximize the effectiveness of hepatitis C prevention and treatment. The research results showed that, compared to the northern region of Anhui, the occurrence of self-selected high-risk behaviors is lower in the central region and higher in the southern region. This may be because the central part of Anhui Province, which includes the capital and neighboring cities, has relatively more economic and educational resources, and people have a higher level of awareness about high-risk behaviors. Therefore, in future prevention and control efforts, greater emphasis should be placed on raising awareness and promoting understanding of self-selected high-risk behaviors in the northern and southern regions of Anhui.

This study should be acknowledged to have several limiting aspects. Firstly, this study was a cross-sectional investigation, which could hinder the inference of causality. Secondly, this survey did not carry out hepatitis C anti-body testing on the survey participants. Therefore, it was hard to conclude whether there is a correlation between the knowledge of hepatitis C prevention and treatment and the occurrence of high-risk behaviors among residents infected with hepatitis C and those not infected with hepatitis C. Finally, participants might tend to give favorable answers during the interview, which was a form of social desirability bias.

## Conclusion

In conclusion, our study indicated that the general population in Anhui Province has low awareness of HCV prevention and treatment and a certain degree of history of high-risk behavior. Recognition of the risk of behaviors that may increase the transmission of the HCV is the key. Therefore, in order to effectively work toward the eradication of HCV, it is essential to strengthen community-level education on hepatitis C prevention and treatment, with particular attention to the older adult and those with low education levels. At the same time, efforts should be intensified in grassroots clinics to raise awareness of hepatitis C prevention and treatment, with a focus on the central and southern regions of Anhui Province. Additionally, education should be enhanced for females, married, divorced or widowed individuals, as well as residents of northern and southern Anhui, to increase their awareness of high-risk behaviors related to hepatitis C and help them avoid such behaviors in daily life. By narrowing the gap between knowledge and behavior, we can contribute to the goal of eliminating hepatitis C by 2030.

## Data Availability

The original contributions presented in the study are included in the article/Supplementary material, further inquiries can be directed to the corresponding authors.
