# Supplementary material for: Awareness of hepatitis C prevention and treatment and high-risk behaviors among the general population in Anhui Province: a cross-sectional study
Source: Front Public Health. 2025 Mar 12;13:1534169. doi: 10.3389/fpubh.2025.1534169 (PMC11936984; doi:10.3389/fpubh.2025.1534169)
Supplement: Supplementary file 1 [file Table_1.docx]

Table 1. The occurrence of self-selected high-risk behaviors for hepatitis C among different characteristics of participants.

| **Characteristics** | Total (n) | Occurrence  n (%) | *χ^2^* | *P*-value |
| --- | --- | --- | --- | --- |
| **Survey area** |  |  | 1.443 | 0.230 |
| Urban area | 1,257 | 477(37.9) |  |  |
| Rural area | 1,166 | 415(35.6) |  |  |
| **Gender** |  |  | 37.904 | <0.001 |
| Male | 1,228 | 379(30.9) |  |  |
| Female | 1,195 | 513(42.9) |  |  |
| **Age group (years)** |  |  |  | <0.001 |
| 15-34 | 913 | 287(31.4) |  |  |
| 35-54 | 1,073 | 420(39.1) |  |  |
| ≥55 | 437 | 185(42.3) |  |  |
| **Marital status** |  |  | 35.055 | <0.001 |
| Single | 474 | 119(25.1) |  |  |
| Married | 1,896 | 750(39.6) |  |  |
| Divorced or widowed | 53 | 23(43.4) |  |  |
| **Residence address** |  |  | 1.946 | 0.163 |
| Anhui Province | 2,391 | 884(37.0) |  |  |
| Other Provinces | 32 | 8(25.0) |  |  |
| **Ethnic Group** |  |  | 2.739 | 0.098 |
| Han | 2,405 | 882(36.7) |  |  |
| Other groups | 18 | 10(55.6) |  |  |
| **Education level** |  |  | 8.159 | <0.043 |
| Primary school and below | 451 | 182(40.4) |  |  |
| Middle school | 785 | 303(38.6) |  |  |
| High school or technical secondary school | 698 | 249(35.7) |  |  |
| College degree or above | 489 | 158(32.3) |  |  |
| **Awareness of hepatitis C** |  |  | 57.933 | <0.001 |
| No | 1,063 | 481(45.2) |  |  |
| Yes | 1,360 | 411(30.2) |  |  |
| **Geographical location** |  |  | 32.732 | <0.001 |
| Northern | 815 | 293(36.0) |  |  |
| Central | 808 | 246(30.4) |  |  |
| Southern | 800 | 353(44.1) |  |  |
